# Supplementary material for: The influence of generative artificial intelligence usage on employees’ innovative job performance
Source: PLoS One. 2026 Jan 22;21(1):e0327786. doi: 10.1371/journal.pone.0327786 (PMC12826493; doi:10.1371/journal.pone.0327786)
Supplement: S1 File — (DOCX) [file pone.0327786.s001.docx]

**Appendix**

Dear Madam/Mr:

Greetings! Thank you very much for taking your valuable time to participate in this questionnaire. It will take about 3 minutes to complete this research. The questionnaire is anonymous and there are no right or wrong questions, so please answer based on your actual situation. Your answers are for academic research purposes only. Thank you again for your participation!

**Instructions:** In recent years, generative AI software (e.g., Doubao, Kimi, Chatgpt, DeepSeek, etc.) has been rapidly growing and penetrating various industries, with far-reaching impacts on work practices. Generative AI software not only helps employees retrieve work information quickly, but also optimizes information sharing by driving collaboration tools and platforms to promote team harmony and efficient collaboration. At the same time, generative AI software assists employees in creative conceptualization, stimulates innovative thinking, and promotes continuous innovation in work results. The wide application of generative AI software technology significantly improves work efficiency, optimizes workflow, provides strong support for team collaboration and innovation, and demonstrates its important value and indispensable role in the work field.

**Screening Questions:** Do you use generative AI software at work?

Yes.

No.(Questionnaire terminated)

| **Please answer the following questions considering the text you read:** | **Strongly Strongly**  **Disagree Agree** | | | | | | |
| --- | --- | --- | --- | --- | --- | --- | --- |
| At work, I create new ideas to achieve improvement. | 1 | 2 | 3 | 4 | 5 | 6 | 7 |
| At work, I mobilize support for new ideas. | 1 | 2 | 3 | 4 | 5 | 6 | 7 |
| I hope to explore innovative ways of working. | 1 | 2 | 3 | 4 | 5 | 6 | 7 |

| **Please answer the following questions considering the text you read:** | **Strongly Strongly**  **Disagree Agree** | | | | | | |
| --- | --- | --- | --- | --- | --- | --- | --- |
| I am quite satisfied with my current job. | 1 | 2 | 3 | 4 | 5 | 6 | 7 |
| Most of the time, I am passionate about my work. | 1 | 2 | 3 | 4 | 5 | 6 | 7 |
| I find that I really enjoy my job. | 1 | 2 | 3 | 4 | 5 | 6 | 7 |
| I have found true joy in my work. | 1 | 2 | 3 | 4 | 5 | 6 | 7 |
| I think my job is quite enjoyable. | 1 | 2 | 3 | 4 | 5 | 6 | 7 |

| **Please answer the following questions considering the text you read:** | **Strongly Strongly**  **Disagree Agree** | | | | | | |
| --- | --- | --- | --- | --- | --- | --- | --- |
| I have learned written knowledge about technology from generative AI software (such as Doubao, KIMI, CHATGPT, DEEPSEEK, etc.). | 1 | 2 | 3 | 4 | 5 | 6 | 7 |
| I have learned knowledge about management skills from generative AI software (such as Doubao, KIMI, CHATGPT, DEEPSEEK, etc.). | 1 | 2 | 3 | 4 | 5 | 6 | 7 |
| I have gained new work experience from generative AI software (such as Doubao, KIMI, CHATGPT, DEEPSEEK, etc.). | 1 | 2 | 3 | 4 | 5 | 6 | 7 |
| I have learned about corporate culture from generative AI software (such as Doubao, KIMI, CHATGPT, DEEPSEEK, etc.). | 1 | 2 | 3 | 4 | 5 | 6 | 7 |

| **Please answer the following questions considering the text you read:** | **Strongly Strongly**  **Disagree Agree** | | | | | | |
| --- | --- | --- | --- | --- | --- | --- | --- |
| I am able to obtain the necessary resources at work to support my new ideas. | 1 | 2 | 3 | 4 | 5 | 6 | 7 |
| When I require additional resources to complete my work, I am usually able to obtain them. | 1 | 2 | 3 | 4 | 5 | 6 | 7 |
| I am able to obtain the resources necessary to perform my work effectively. | 1 | 2 | 3 | 4 | 5 | 6 | 7 |

| **Please answer the following questions considering the text you read:** | **Strongly Strongly**  **Disagree Agree** | | | | | | |
| --- | --- | --- | --- | --- | --- | --- | --- |
| I use generative AI software (such as Doubao, KIMI, ChatGPT, DEEPSEEK, etc.) to collect work-related information and share content. | 1 | 2 | 3 | 4 | 5 | 6 | 7 |
| I use generative AI software (such as Doubao, KIMI, CHATGPT, DEEPSEEK, etc.) to disseminate content at work. | 1 | 2 | 3 | 4 | 5 | 6 | 7 |
| I access content created by others using generative AI software (such as Doubao, KIMI, ChatGPT, DeepSeek, etc.) | 1 | 2 | 3 | 4 | 5 | 6 | 7 |

| **Please answer the following questions considering the text you read:** | **Strongly Strongly**  **Disagree Agree** | | | | | | |
| --- | --- | --- | --- | --- | --- | --- | --- |
| I use generative AI software (such as Doubao, KIMI, CHATGPT, DEEPSEEK, etc.) to build new relationships at work. | 1 | 2 | 3 | 4 | 5 | 6 | 7 |
| I use generative AI software (such as Doubao, KIMI, CHATGPT, DEEPSEEK, etc.) to connect with people I wouldn't meet at work. | 1 | 2 | 3 | 4 | 5 | 6 | 7 |
| I use generative AI software (such as Doubao, KIMI, CHATGPT, DEEPSEEK, etc.) to maintain close social connections with colleagues at work. | 1 | 2 | 3 | 4 | 5 | 6 | 7 |
| I make like-minded friends through generative AI software (such as Doubao, KIMI, CHATGPT, DEEPSEEK, etc.). | 1 | 2 | 3 | 4 | 5 | 6 | 7 |
| I use generative AI software (such as Doubao, KIMI, CHATGPT, DEEPSEEK, etc.) to find friends who share my interests. | 1 | 2 | 3 | 4 | 5 | 6 | 7 |

**Questions of Demographics**

1.What is your gender?  Male  Female

2.What is your age in years?

Under 25 years old

26–35

36 and above

3.Please indicate your educational background.

High school and below

Junior college

Undergraduate degree

Master degree or above

4.Marital status? Married Spinsterhood
