# Supplementary material for: The influence of generative artificial intelligence usage on employees’ innovative job performance
Source: PLoS One. 2026 Jan 22;21(1):e0327786. doi: 10.1371/journal.pone.0327786 (PMC12826493; doi:10.1371/journal.pone.0327786)
Supplement: Attachment — Submitted filename: REVIEW REPORT.docx [file pone.0327786.s003.docx]

**REVIEW REPORT**

Manuscript Number: PONE-D-25-16015

Article Type: Research Article

Full Title: The Influence of Using Generative Artificial Intelligence Tools on Employee's Innovative Job Performance

Short Title: The Influence of Using Generative Artificial Intelligence Tools on Employee's Innovative Job Performance

Corresponding Author: Ayuan Zhang Anhui Normal University wuhu, CHINA

Date: 2025-04-18

**General Evaluation**

The manuscript proposes a model that explains the effects of cognitive and social usage patterns of generative artificial intelligence (GenAI) tools on employees' knowledge transfer, resource acquisition, job satisfaction and innovative job performance. The paper is based on survey data from 366 Chinese employees and analyzed with SmartPLS. The model seems to be theoretically consistent and empirically supported.

## However, the language, conceptual presentation and interpretation of the study needs some corrections and significant improvement. Especially the sections that seem to be sourced from Google Translate distort the meaning and reduce the quality of academic expression.

## Overall, while the study addresses a timely and relevant research problem, it requires substantial revisions in language, statistical reporting, and conceptual elaboration before it can be considered for publication.

## Introduction

1. Language problems and repetitive expressions reduce comprehensibility.

Some sentences in the introduction need to be revised. For example:

“Tools influence employees' innovative work performance by creating an overlapping boundary between work tasks and personal space.”

Similar statements are repeated with minor changes throughout the introduction, leading to unnecessary redundancies.

2. The research gap is not clearly stated.

Although the topic is topical, the introduction does not clearly state what gap in the literature this study fills. The uniqueness of this research could be emphasized. For example, the Use and Satisfaction Theory (UST) could be briefly introduced and information on its application in organizational settings could be added.

**Recommendation:** The authors should state more clearly what is missing from existing research and how their model advances theoretical or empirical understanding in the field.

**Evaluation of the Theoretical Framework and Hypotheses**

1. The explanation of the Uses and Gratifications Theory (UGT) is overly long and not well integrated with the research context.

The text includes general definitions of UGT such as:

“The uses and gratification theory helps understand how individuals actively seek out specific media and content to satisfy particular needs.”

However, it is not clear how these descriptions relate to the cognitive and social use of productive AI tools in the workplace. This section should be simplified, and the theoretical framework should be directly related to the research variables.

2. Some hypotheses lack strong theoretical justification.

For example, hypothesis H2 is presented as follows:

“H2: The social use of GenAI tools positively impacts resource acquisition.”

However, the direct relationship between social use and resource acquisition is not sufficiently explained. What kind of social interactions are linked to resource acquisition or how GenAI tools support these processes is not supported by literature. This relationship seems like an artificial connection and leaves the reader with a sense of theoretical emptiness.

**Research Model and Evaluation of Methodology**

Can the research model be presented in the preliminary part of the study (suggestion only)?

Can indirect relationships (if any) be expressed more clearly?

There is no explicit statement on ethical approval. This situation can be explained.

A little more information about the pilot study could be added.

**Result**

Although indirect effects are conceptually included in the model, they have not been statistically tested. Overall, this section needs to be reported more systematically and linked more deeply to the theoretical context.

**Discussion, Conclusions and Contributions**

The discussion section mostly repeats the findings. An analytical level comparison with the existing literature would compensate for this. The theoretical and practical contributions are stated in very general terms and the unique aspects of the study are not clarified. Could at least one sentence be added?

The limitations section remains superficial, and could it be expanded? Can the cultural context be emphasized (just a suggestion)?
