# Supplementary material for: The influence of generative artificial intelligence usage on employees’ innovative job performance
Source: PLoS One. 2026 Jan 22;21(1):e0327786. doi: 10.1371/journal.pone.0327786 (PMC12826493; doi:10.1371/journal.pone.0327786)
Supplement: Attachment — Submitted filename: Response to Reviewers.docx [file pone.0327786.s005.docx]

**Cover letter**

**Dear Reviewers:**

**Thank you for your letter and for the reviewers’ comments concerning our manuscript entitled “The Influence of Using Generative Artificial Intelligence Tools on Employee's Innovative Job Performance” (ID: PONE-D-25-16015). Those comments are all valuable and very helpful for revising and improving our paper, as well as the important guiding significance to our researches. We have studied comments carefully and have made correction which we hope meet with approval. Revised portion are marked with different colors in the paper. The main corrections in the paper and the responds to the reviewer’s comments are as flowing:**

**Reviewer #1:**

1. **The Uses and Gratifications Theory (UGT) has been addressed extensively. The classification of motivations within this theory is explained at great length; it is recommended that this section be restructured more concisely and directly connected to the research model. Additionally, the relationship between UGT’s media-based origins and the context of AI use should be discussed more clearly and critically.**

**Answer：**We sincerely appreciate the reviewer's valuable feedback. In response to the comments, we have revised the section on the Uses and Gratifications Theory (UGT) as follows part: first, We have introduced the core concepts of UGT, Subsequently, the research of this theory in the field of employee performance is briefly discussed. Finally, it was found that previous scholarly research has mostly focused on examining the impact of using various social media tools and technologies at work on employee innovativeness, and no scholars have yet applied the theory to categorize the motivation for the use of generative AI tools, which is therefore a research gap for this paper.

**These revisions have been highlighted in blue in the revised manuscript.**

“Most previous research on GenAI has treated user behavior toward these tools as a singular, homogeneous pattern. However, as technology evolves—particularly tools like GenAI that support a range of applications—this broad approach fails to capture the complexity and multidimensional nature of user behavior. Thus, there is a need for a more nuanced perspective that explores the diverse behavioral patterns of GenAI usage and the mechanisms that influence them across different contexts (Barki et al., 2007; Burton-Jones & Straub, 2006; Jasperson et al., 2005; Orlikowski & Iacono, 2001; Venkatesh et al., 2008). Drawing on UGT, this framework establishes an intrinsic link between users' behavioral choices and their outcomes, making it ideal for examining the motivations behind GenAI adoption and its influence mechanisms in the workplace (Lometti et al., 1977; Stafford et al., 2004). Accordingly, this paper aims to systematically categorize and analyze the specific behavioral patterns associated with employees' use of GenAI tools in work settings, focusing on their intrinsic motivations for adopting these technologies.”

2. **The current model focuses solely on cognitive and social motivations. However, other important components of UGT, such as hedonic motivation, have been excluded. This creates a lack of full alignment with the theory. The model should be expanded with new hypotheses to address this gap.**

**Answer：**We sincerely appreciate the reviewer’s insightful feedback. As rightly noted, the traditional Uses and Gratifications Theory (UGT) categorizes media usage motivations into three primary types: social, hedonic, and cognitive (Ali-Hassan et al., 2015). While our study acknowledges this foundational framework in discussing generative AI tool usage motivations, we deliberately focused on social and cognitive motivations for the following reasons: GenAI tools differ from social media tools in terms of their usage effects; for social media, one of the main uses of social media is hedonic, i.e., to enjoy the experience of relaxation, pleasure, etc., brought by social media, e.g., Ali-Hassan et al. (2015) classified people's motivations for using social media in workplaces as social motivation, hedonic motivation and cognitive motives; however, for generative AI tools, people use this tool in the workplace more for information search, communication and delivery, and less for entertainment or enjoyment. In addition, some scholars have also pointed out that social and cognitive motivation have a direct correlation with the work context (Zhang et al., 2018). Therefore, hedonic motivation was not used in this paper for this study, but the reviewer's comments are greatly appreciated, and this is the direction of our future research; in the future, we can consider scenarios in which people use generative AI tools for hedonic purposes and discuss how this hedonic motivation will affect people's behaviors in that scenario.

These revisions have been highlighted in blue in the revised manuscript.

“The original UGT provides a foundation for understanding media engagement motivations, which are typically categorized into three key dimensions: social, hedonic, and cognitive motivations (Ali-Hassan et al., 2015). Social motivation refers to the use of media to connect with others who share similar interests or topics (Papacharissi & Mendelson, 2011). Hedonic motivation is driven by the desire to maximize leisure time through enjoyable media experiences, such as optimizing relaxation (Quan-Haase & Young, 2010), alleviating occupational stress (Papacharissi & Mendelson, 2011), and engaging in recreational rejuvenation (Shao, 2009). Cognitive motivation encompasses two key aspects: collaborative knowledge development, which often relies on digital platforms like WeChat (Raacke & Bonds-Raacke, 2008; Shao, 2009), and the utilization of media for learning and development purposes. According to Skjuve et al. (2024), people primarily use GenAI tools to improve productivity, foster creativity, and engage in social interactions, learning, and development. Some scholars suggest that social and cognitive motivations are directly linked to the work context. For example, Zhang et al. (2018) distinguished between social and task-related motivations for social media use. While social media use primarily influences personal interactions, the role of GenAI in the workplace is more closely tied to knowledge management (Chen et al., 2021), innovation stimulation, remote work support (Papacharissi & Mendelson, 2011), and social collaboration (Raacke & Bonds-Raacke, 2008; Quan-Haase & Young, 2010; Chen et al., 2024). In contrast, GenAI has not been shown to significantly affect employees' relaxation or time-killing behaviors. Building on these insights, this study argues that employees’ motives for using GenAI tools in the workplace can be categorized into "cognitive" and "social" motives. Consequently, this study proposes that employees' behaviors when using GenAI tools can be further classified into two distinct types: cognitive-based GenAI use behaviors and social-based GenAI use behaviors. The former involves sharing relevant knowledge and accessing or understanding information provided by others through GenAI tools (Shao, 2009; Raacke & Bonds-Raacke, 2008), while the latter pertains to using GenAI tools to find partners with shared interests, establish new social relationships, and expand social networks (Papacharissi & Mendelson, 2011).”

3. **Although the study is based on cross-sectional data, it makes causal claims. This is a significant methodological issue. The authors should either soften the causal language or propose longitudinal or experimental studies to support such claims.**

**Answer：**We sincerely appreciate the reviewers' comments. In response, we have strengthened the empirical analysis by incorporating robustness checks.

In the limitations and future research section, we supplemented our research agenda: "To address the causal inference limitations inherent in cross-sectional data, we plan to conduct quasi-natural experiments with partner organizations, comparing changes in employee innovation behaviors before and after AI tool implementation, with control groups using traditional knowledge management systems."

These revisions have been highlighted in blue in the revised manuscript.

“Finally, regarding research design, the study primarily relied on homogeneous data sources, specifically employee self-assessments, which may introduce common methodological biases. To improve the robustness of future findings, multi-source data collection methods should be considered, such as incorporating supervisor or coworker evaluations, or secondary data. Additionally, hierarchical linear modeling or multilevel analysis could be employed to test the research hypotheses. In conclusion, while this study highlights the facilitating effect of GenAI tools on innovative job performance, it has limitations in research design, content, and subject matter. Future studies can expand the understanding of GenAI tool applications in organizations by utilizing multi-source data, adopting longitudinal research designs, broadening research content, and examining individual differences, thereby providing more comprehensive support for both theoretical development and practical applications.”

4. **The contribution to the literature section repeats many concepts. Does this study merely apply the Uses and Gratifications Theory, or does it extend it? The claim of theoretical contribution should be strengthened.**

**Answer：**We sincerely appreciate the reviewers’ feedback. This study primarily applies the Uses and Gratifications Theory (UGT) to classify employees’ motivations for adopting generative AI tools in workplace settings, constituting its primary theoretical contribution.

To reinforce this contribution, we have revised the opening subsection of the Theoretical Contributions section to explicitly emphasize how this research extends UGT’s domain of application. Traditionally rooted in communication studies, UGT has only recently been extended to contexts such as employee entrepreneurship and social media (Katz et al., 1973；Ali-Hassan et al., 2015; Zhang et al., 2018). This study further expands the UGT framework to encompass generative AI adoption in professional environments. This extension not only significantly enriches the theory’s applicability but also provides a novel theoretical lens for examining how generative AI tools shape employee behaviors and decision-making processes.
These revisions have been highlighted in blue in the revised manuscript.

“First, this study broadens the scope of UGT, which has traditionally been applied primarily in the field of communication. Only in recent years has its application expanded to areas such as employee entrepreneurship and social media (Katz et al., 1973; Ali-Hassan et al., 2015; Zhang et al., 2018). This paper further extends the UGT framework to explore the use of GenAI tools in workplace contexts, empirically examining how cognitive and social motivations influence employees' use of these tools by enhancing their knowledge transfer and resource acquisition capabilities. In turn, this impacts their job satisfaction and innovative job performance. This extension not only significantly enriches the theoretical application of UGT but also offers a fresh perspective on understanding the mechanisms by which GenAI tools shape employee behavior.”

5. **The translation and adaptation process of the scales is described in a very superficial manner. The use of GenAI tools can vary across cultural contexts, particularly in China. The appropriateness of the scales for the Chinese cultural context should be supported with stronger justification.**

**Answer：**We sincerely appreciate the reviewers’ insightful feedback and fully concur with the critical observation regarding the need for more systematic cultural adaptation in our scale localization, particularly given China’s unique generative artificial intelligence (GenAI) usage context. To address this, we emphasize that the original scale development already incorporated adjustments for cross-cultural differences in GenAI tool utilization, with deliberate wording modifications reflecting these considerations. For the reviewers’ convenience, we have included annotated excerpts from the original questionnaire (see screenshot below) to visually demonstrate our cultural adaptation efforts. The screenshot shows that:

The questionnaire explicitly defines GenAI tools at the outset. It lists China-specific GenAI platforms commonly used by Chinese professionals, including ChatGPT, KIMI, Doubao, and DeepSeek, aligning with local usage patterns

Furthermore, we have strengthened the methodological transparency in the revised manuscript’s Variable Measurement section by explicitly stating:"All measurement items were rigorously adapted to account for Chinese users’ distinctive GenAI adoption habits and workplace dynamics.

These revisions have been highlighted in blue in the revised manuscript.

6. **In the structural model, the direct effect of social use on innovative performance appears negative and insignificant. This result has been overlooked by the authors. It should be seriously considered, discussed, or the rationale for including this variable in the model should be re-evaluated.**

**Answer：** We sincerely appreciate the reviewers' valuable feedback and fully acknowledge the identified issue. In response, we have re-examined the mediating pathways using SmartPLS 4.0 with 5,000 Bootstrap resampling iterations. The results (as shown in the fourth pathway of the results table) demonstrate that: Socially motivated GenAI tool usage exerts a statistically significant positive indirect effect on innovation performance through the dual mediation of resource acquisition and job satisfaction, with an indirect effect value of 0.149 (p < 0.001). This finding indicates that while the direct pathway remains non-significant, a crucial mediating mechanism exists in explaining how GenAI adoption influences workplace outcomes.

These revisions have been highlighted in blue in the revised manuscript.

**Table 7. Mediator analysis**

| Hypotheses | Indirect effects | T-value | Confidence  Interval | | P |
| --- | --- | --- | --- | --- | --- |
|  |  |  | Low | Up |  |
| Cu→Kno_tra→Job_Stf→I_Job_Pf | 0.139 | 5.342 | 0.093 | 0.195 | 0.000 |
| Cu→Kno_tra→Job_Stf | 0.249 | 6.447 | 0.175 | 0.328 | 0.000 |
| Kno_tra→Job_Stf→I_Job_Pf | 0.201 | 5.678 | 0.138 | 0.275 | 0.000 |
| **Su→Acq_res→Job_Stf→I_Job_Pf** | **0.149** | **6.263** | **0.106** | **0.200** | **0.000** |
| Su→Acq_res→Job_Stf | 0.269 | 7.155 | 0.197 | 0.345 | 0.000 |
| Acq_res→Job_Stf→I_Job_Pf | 0.268 | 7.982 | 0.204 | 0.336 | 0.000 |

7. **90% of participants have an undergraduate or higher level of education. The implications of this for the generalizability of the results should be discussed, as this sample may not represent the broader Chinese workforce.**

**Answer：**We sincerely appreciate the reviewers’ constructive feedback and fully acknowledge the identified concern. This study was published on the Credamo platform, and this sample distribution may be explained by the strong correlation between education and the nature of the work. In this paper, participants were asked at the beginning of the questionnaire whether they had used generative AI tools in the workplace, and those who chose “no” would not participate in the follow-up questionnaire. For those who chose “yes”, the main use of generative AI tools in the workplace is for knowledge management, and this group may belong to the higher education group, thus resulting in the above distribution. We have discussed the generalizability of the sample in the “**Limitations and Future Directions” section** of the revised paper, suggesting that the sample data may not be generalizable, which is a shortcoming of this paper, and that future research will delve deeper into different educational backgrounds.

These revisions have been highlighted in blue in the revised manuscript.

“First, regarding the research sample, the data predominantly reflects general enterprise employees, with 90% holding an undergraduate degree or higher. This sample does not sufficiently account for the diverse application scenarios of GenAI tools across various educational levels, industries, occupations, and managerial tiers. Future research could examine the differential effects of GenAI tools on employee performance across specific industries (e.g., technology, finance, healthcare), management levels (e.g., frontline employees, middle managers, senior leaders), and geographical contexts (e.g., Tier 1, Tier 2, Tier 3, and Tier 4 cities). Furthermore, individual difference factors, such as employees' educational background, personality traits (e.g., openness, conscientiousness), and work habits, may moderate the impact of GenAI tool usage. Future studies should investigate how these individual differences influence the relationship between GenAI tool use and employee innovative job performance (or other performance metrics), thereby providing a theoretical foundation for organizations to devise differentiated technology usage strategies.”

8. **The limitations section at the end of the paper is overly general. The effects of issues such as the ethical use of GenAI tools, privacy concerns, and algorithmic bias should be taken into account.**

**Answer：**Thank you very much for the reviewers' comments and for recognizing the research value of this paper and your insightful suggestions for revision. We fully agree with you that the limitations section needs to deepen the analysis of key topics such as ethics, privacy, and algorithmic bias.

Based on your suggestion, **we have revised the “Research Limitations and Future Directions” section of the original paper,** and rewritten the section on shortcomings and outlook, regarding the failure to adequately consider the differentiated application scenarios of generative AI tools for research participants across different education, industry, occupation, and management levels. As well as the fact that the results of the study were all positive effects, the negative effects of generative AI on job performance will be considered in the future, and the fact that there was a single source of data, relying mainly on employee self-assessment data.

These revisions have been highlighted in blue in the revised manuscript.

“Second, this study observed the positive indirect effects of GenAI tool use, driven by cognitive and social motivations, on innovative job performance. However, it did not explore potential negative or attritional effects. While positive outcomes such as knowledge exchange and resource acquisition were noted, the study did not quantitatively assess potential downsides, such as attention dissipation (e.g., frequent discussions without productive outcomes) or information overload (e.g., receiving a large volume of unstructured input) resulting from excessive social interactions. This unidimensional view of resource acquisition may obscure the inhibitory effects of "social overload" on innovative thinking. Future research could address the dual impact of GenAI tools used for social purposes on employee behavior (e.g., job satisfaction and performance), exploring both positive and negative outcomes.”

9. **Data availability is stated as “available upon request,” but PLOS ONE requires an open data policy. The authors should clearly specify under what conditions and on which platform the data will be shared.**

**Answer：**We sincerely appreciate the reviewer’s guidance on aligning with PLOS ONE’s open data policy. We hereby clarify our data sharing protocol as follows:

Upon acceptance of the manuscript, all de-identified raw data and data supporting the results of this study will be deposited in public databases, along with the repository name, link to the data, and a unique identifier in the statement to ensure full compliance with PLOS ONE's open access requirements.

10. **The literature heavily relies on Western-centric references. More up-to-date local sources and case studies specific to the Chinese workforce should be included.**

**Answer：**We sincerely thank the reviewers for their valuable suggestions and fully concur with the observation regarding the insufficient attention to China-specific contexts in existing literature. In response, we have made the following additions and improvements:

In the research hypotheses section, we have added some local Chinese literature, which mainly focuses on the discussions of “cognitively motivated generative AI tool use and knowledge transfer”, “socially motivated generative AI tool use and resource acquisition ability”, “knowledge transfer, resource acquisition ability and job satisfaction”, and “social motivation”, “knowledge transfer, resource acquisition ability and job satisfaction”, and ‘job satisfaction and employee innovative job performance’, with specific additions by Zou et al. (2023), Mu et al. (2023), Zhan and Li (2024), and Miao et al. (2024), etc. Through the above adjustments, the number of Chinese local literatures was increased from 4 to 18, which will significantly enhance the explanatory power of the research findings on the local management phenomena in China, and provide a unique oriental perspective for the study of global management.

These revisions have been highlighted in blue in the revised manuscript.

“3.1 Cognitive use of GenAI tools and knowledge transfer

Knowledge transfer refers to the intentional exchange of explicit and implicit expertise between different organizational entities, facilitated by various communication channels (Spreitzer, 1996). Knowledge management frameworks illustrate that cognitive GenAI adoption accelerates knowledge acquisition in two key ways: by speeding up transactional processes and enhancing cognitive absorption (Autio & Nambisan, 2018). GenAI systems optimize the handling of informational repositories through algorithms that filter, semantically summarize, and retrieve content contextually (Gindert & Müller, 2024). For instance, Morgan Stanley utilized GPT-4 to process 100,000 company-specific investment documents and business materials, refining their knowledge assistance system for financial advisors (Gindert & Müller, 2024). Digital interfaces powered by GenAI technology transform complex information systems into more accessible, knowledge-friendly formats using adaptive data segmentation and connection methods (Autio & Nambisan, 2018). In practical applications, when employees engage in online meetings, GenAI tools can help generate meeting minutes, extract summaries from video or audio recordings, and organize key content—an application widely used on platforms such as ZOOM. Based on knowledge management theory, Zou et al. (2023) found that GenAI tools help break down interdepartmental knowledge barriers, fostering cross-disciplinary creative integration and enabling breakthrough solutions. Mu et al. (2023) further demonstrated that AI-assisted brainstorming tools significantly enhance team innovation output by generating diverse creative solutions. Thus, the following hypothesis is proposed:

H1: Cognitive use of GenAI tools positively impacts employees' knowledge transfer.

3.2 Social use of GenAI tools and resource acquisition capabilities

Workers require resource acquisition for their tasks, which includes access to information, materials, office space, and adequate working time. The acquisition of valuable resources within an organization originates from both internal elements and external sources, such as customers, suppliers, competitors, universities, government agencies, and technology intermediaries (Chen and Yang, 2014). The advent of GenAI tools enables the formation of informal relationship networks, in line with social exchange theory, by connecting individuals with shared interests and backgrounds, thereby expanding users' social networks (Russell and Norvig, 2002; Turner and Kuczynski, 2019). Organizational members are encouraged to promote collaborative tools, social sharing platforms, and interactive communication channels, as these facilitate team connections, enhance productive teamwork, and foster a sense of belonging among employees. GenAI tools, when utilized for social motivation, enable employees to access essential knowledge resources, reducing the strain on their personal resource usage. Noy and Zhang (2023) demonstrated that ChatGPT users experienced increased writing efficiency while significantly reducing their workplace time consumption. Jia et al. (2023) observed that AI technology implementation leads to better work distribution and increased employee creativity by alleviating human resource constraints. Zhan and Li (2024) highlighted that the informational advantage in a generative environment greatly enhances cognitive resource efficiency and situational awareness. Through GenAI tools, employees can more easily engage in communication, share information both within and outside the organization, and strengthen emotional connections with internal and external stakeholders. These social networks—comprising various internal and external members of the enterprise—provide a foundation of trust that supports employees' resource acquisition efforts, enhancing their ability to secure resources (Liu et al., 2019; Miao et al., 2024). Based on these insights, the following hypothesis is proposed:

H2: Social use of GenAI tools positively impacts employees' resource acquisition ability.

3.3 Knowledge transfer, resource acquisition, and job satisfaction

As previously discussed, knowledge transfer refers to the sharing of knowledge across organizational boundaries or between individuals, encompassing the dissemination of knowledge in various forms within or across different entities (Szulanski, 1996). Resource acquisition ability highlights employees' capacity to easily access relevant resources that facilitate their job tasks (Spreitzer, 1996). Employee job satisfaction is a multifaceted evaluation of work, representing the positive emotional state derived from the assessment of job experiences (Locke, 1976).

Knowledge transfer is a critical component of knowledge management, contributing to enhanced employee job satisfaction (Kianto et al., 2016; Zhou & Tang, 2017; Usmanova et al., 2021; Fischer & Döring, 2022). Knowledge transfer encourages employees to voluntarily share their work experiences, skills, and other job-related knowledge, which improves work efficiency (Usmanova et al., 2021; Fischer & Döring, 2022). For instance, Usmanova et al. (2021) found that knowledge-sharing behaviors among skilled employees in IT companies significantly contribute to their job satisfaction. Additionally, fostering a corporate culture that promotes knowledge transfer helps create a supportive interpersonal environment, strengthening work relationships and, consequently, enhancing job satisfaction (Borman & Motowidlo, 1997; Misuraca, 2013; Zhang et al., 2023). Research also suggests that knowledge sharing and transfer within organizations generate opportunities for concept development and job-related information acquisition, leading to greater job satisfaction (Misuraca, 2013; Zhou & Wei, 2011). Job satisfaction increases as knowledge transfer practices become more accessible within organizational environments, as indicated by Varshney and Damanhouri (2013). Based on these insights, the following hypothesis is proposed:

H3: Knowledge transfer positively impacts the improvement of employees' job satisfaction.

Previous research has established a positive relationship between resource acquisition capabilities and employees' job satisfaction (Mohr & Zoghi, 2008; Zatzick, 2011). Employees' resource acquisition ability is specifically demonstrated in their capacity to solve work-related problems, such as accessing external, tacit knowledge, obtaining additional resources to complete tasks, and other related factors (Spreitzer, 1996). A higher level of resource acquisition ability indicates that employees are more likely to enter a productive work state, maintain greater concentration, and exhibit enhanced cognition and assimilation of the organization's objectives, which ultimately leads to higher job satisfaction (Zatzick, 2011; Cheng & Wu, 2023; Xie et al., 2021). When employees adopt GenAI tools with an openness to change and innovation, coupled with a positive attitude, they can foster effective collaboration, strengthen their sense of identity and well-being, and improve their job satisfaction (Wang & Yao, 2022; Yin & Niu, 2024; Wang, 2024; Gayathri & Bella, 2024). For instance, Wang (2024) found that GenAI’s automated knowledge integration function reduces employees' information overload, allowing them to focus more on core tasks and, consequently, increasing job satisfaction. Gayathri & Bella (2024) highlighted that GenAI enhances employees' ability to quickly access both internal and external organizational resources. GenAI tools applied to social media platforms support content generation or improve user experiences, thereby boosting employees' familiarity with their work, improving their task completion abilities, and fostering more positive work attitudes, which contribute to greater job satisfaction. Based on these insights, the following hypothesis is proposed:

H4: Employee resource acquisition positively impacts the improvement of job satisfaction.

3.4 Job satisfaction and employees’ innovative job performance

Innovative job performance refers to employees’ work activities that go beyond conventional expectations, including the generation of creative ideas, problem-solving approaches, and the identification of new solutions (Katz, 1964; Scott & Bruce, 1994; Amabile et al., 1996). For businesses to thrive, enhancing competitiveness is critical, and competitiveness is driven by innovation. Thus, employees' innovative job performance is a core element in boosting organizational competitiveness (Yu & Shi, 2006; Liu & Li, 2025).

Extensive research indicates that high job satisfaction directly contributes to enhanced innovative performance. First, when employees are satisfied with their jobs, they tend to enjoy their work and exhibit greater willingness to perform (Boss, 1978). For instance, Schappe (1998) highlighted that higher job satisfaction leads to more positive perceptions of the organization, better work performance, and greater willingness to engage in behaviors beneficial to the organization. Xiao (2020) found that highly satisfied employees are more likely to internalize organizational goals, which in turn improves their innovative performance through organizational commitment. Second, job satisfaction is strongly and positively linked to the organizational innovation climate, which in turn positively influences employee innovation performance (Wang, 2011; Pang & Shi, 2016; Miao et al., 2024). For example, Yan and Bai (2016) found that a high-performance work system fosters a positive innovation climate, which enhances job satisfaction and boosts innovative behaviors. Finally, job satisfaction impacts employees' innovative job performance by influencing their emotional state, which affects their motivation and creativity (Amabile, 1988; Zhou & George, 2001; Zou et al., 2015). Amabile (1988) argued that positive affective states, such as job satisfaction, enhance cognitive flexibility and willingness to take risks, thereby increasing innovative output. Zhou and George (2001) suggested that job satisfaction indirectly promotes innovative behaviors by enhancing intrinsic motivation, such as interest in the task itself. Based on these findings, the following hypothesis is proposed:

H5: Employees' job satisfaction positively impacts the improvement of their innovative job performance.”

**Reviewer #2**

1. **Reporting of statistical analyses is incomplete. In the Results section, important indicators such as beta coefficients, p-values, R² are not given in full in some places. I did not see that indirect effects (mediation) were tested. The overall explanatory power of the model could be expanded.**

**Answer：**We sincerely appreciate the reviewer’s meticulous evaluation of our methodological rigor. To address the concerns regarding incomplete statistical reporting, we have implemented systematic revisions to enhance the transparency and interpretability of our results:

1） Supplementation of Key Statistical Indicators: Table 4 now includes standardized β coefficients with corresponding significance levels (p-values) for all pathways. Figure 2 (the structural model diagram generated via SmartPLS 4.0) explicitly displays the R² values for endogenous variables to quantify explanatory power. Table 7 comprehensively reports specific indirect effect tests validated through the Bias-Corrected Bootstrap method (5,000 resamples) to confirm mediation pathways.
2） Visual Optimization: All tables have been standardized to include full statistical reporting requirements (e.g., confidence intervals, effect sizes). Figure 2 has been redesigned for clarity, with enhanced node labeling and pathway visualization to improve the interpretability of SmartPLS outputs.

These revisions have been highlighted in blue in the revised manuscript.

**Table 4. Path Coefficients and Significant Levels**

| **Path** | **β** | **t** | **95% confidence interval** | **p** | **Hypothesis** | **f²** | **Effect size** |
| --- | --- | --- | --- | --- | --- | --- | --- |
| CU→know_tra | **0.689** | 23.726 | [0.631, 0.746] | **0.000** | H1 | 0.906 | Large |
| SU→Acq_res | **0.558** | 13.093 | [0.473, 0.639] | **0.000** | H2 | 0.453 | Large |
| know_tra→Job_Stf | **0.361** | 6.760 | [0.259,0.470] | **0.000** | H3 | 0.185 | Medium |
| Acq_res→Job_Stf | **0.481** | 9.612 | [0.380, 0.576] | **0.000** | H4 | 0.328 | Medium |
| Job_Stf→I_Job_P | **0.556** | 14.421 | [0.483, 0.632] | **0.000** | H5 | 0.448 | Large |

**Fig2. Results of structural equation modelin**

**Table 7. Mediator analysis**

| Hypotheses | **Indirect effects** | T-value | Confidence  Interval | | **P** |
| --- | --- | --- | --- | --- | --- |
|  |  |  | Low | Up |  |
| Cu→Kno_tra→Job_Stf→I_Job_Pf | **0.139** | 5.342 | 0.093 | 0.195 | **0.000** |
| Cu→Kno_tra→Job_Stf | **0.249** | 6.447 | 0.175 | 0.328 | **0.000** |
| Kno_tra→Job_Stf→I_Job_Pf | **0.201** | 5.678 | 0.138 | 0.275 | **0.000** |
| Su→Acq_res→Job_Stf→I_Job_Pf | **0.149** | 6.263 | 0.106 | 0.200 | **0.000** |
| Su→Acq_res→Job_Stf | **0.269** | 7.155 | 0.197 | 0.345 | **0.000** |
| Acq_res→Job_Stf→I_Job_Pf | **0.268** | 7.982 | 0.204 | 0.336 | **0.000** |

“The data indicate that the indirect effect of using GenAI tools for cognitive purposes on innovative job performance, mediated through knowledge transfer and job satisfaction, is significant (β = 0.139, p < 0.001), suggesting a mediating effect. Additionally, knowledge transfer mediated the impact of cognitive-purpose GenAI tool use on job satisfaction (β = 0.249, p < 0.001), and job satisfaction, in turn, mediated the relationship between knowledge transfer and innovative job performance (β = 0.201, p < 0.001). Similarly, the use of GenAI tools for social purposes showed a significant indirect effect on innovative job performance through resource acquisition and job satisfaction (β = 0.149, p < 0.001). Moreover, resource acquisition mediated the effect of social-purpose GenAI tools on job satisfaction (β = 0.269, p < 0.001), while job satisfaction mediated the impact of resource acquisition on innovative job performance (β = 0.268, p < 0.001).”

2. **Discussion section could be organized. The findings are only repeated and the linking and comparison with the literature is weak. This section could be expanded to discuss similarities or differences in findings with previous studies.**

**Answer：**We sincerely appreciate the reviewer’s constructive critique of the discussion section and fully acknowledge the issues of redundant findings and insufficient scholarly dialogue. In the revised manuscript, we have restructured the discussion as follows:

1) Deleted the original results repetition. In the original text, we found that two paragraphs of results discussion were repetitive, so we merged and deleted the above results content.

2) In the discussion of results, the findings of this paper, i.e., the mechanism of the impact of employees' use of generative AI tools based on different motivations on their innovative job performance, are first elaborated in detail. Based on the findings of the study and examples, the impact of cognitive and social motivations on the transfer of knowledge and acquisition of resources, respectively, and how this affects the job satisfaction, which ultimately promotes the innovative job performance of the employees, are explained in detail. job performance.

3) In the theoretical contribution section, we “expand the research field of use and satisfaction theory”, “enrich the research perspective of the impact of generative AI tools on employee behavior”, and “validate employees' knowledge sharing ability, resource acquisition ability, and employee satisfaction”. We discuss this paper from the perspectives of “expanding the research field of use and satisfaction theory”, “enriching the research perspective of the impact of generative AI tools on employee behavior”, and “verifying the intrinsic mechanism of employee knowledge-sharing ability and resource acquisition ability as well as employee satisfaction”, and we strengthen the theoretical contributions of this paper by combining the previous literatures.

These revisions have been highlighted in blue in the revised manuscript.

“6. General discussion

This study employed UGT to investigate the impact of employees' use of GenAI tools on their innovative job performance, driven by different motivations. The analysis revealed that both cognitively and socially motivated use of GenAI tools significantly enhances innovative job performance. Specifically, cognitively motivated use of GenAI tools promotes knowledge transfer behaviors. For instance, employees' cognitive use of these tools optimizes the knowledge management process, reduces their workload, and boosts the efficiency of knowledge transfer. This not only strengthens employees' professional capabilities but also lays a solid knowledge foundation for their innovative behaviors. On the other hand, the socially motivated use of GenAI tools significantly enhances employees' knowledge acquisition abilities. For example, employees can use these tools to share experiences, discuss challenges, or seek help from colleagues, thereby fostering cross-departmental collaboration and gaining access to additional work resources. These two pathways—cognitive and social—contribute to increased job satisfaction, which subsequently boosts innovative job performance. More specifically, the use of GenAI tools driven by cognitive and social motivations indirectly enhances job satisfaction by improving knowledge transfer and knowledge acquisition. On one hand, efficient knowledge transfer enables employees to acquire new skills and knowledge more quickly, leading to a sense of achievement and satisfaction with their work. On the other hand, enhanced access to resources provides employees with more opportunities for development and growth, fostering confidence and optimism about their career prospects. These positive work experiences collectively enhance employee satisfaction. Satisfied employees are more likely to invest greater effort and time into their work, demonstrating higher motivation and creativity, which, in turn, fosters improved innovative work performance.

6.1 Theoretical contributions

First, this study broadens the scope of UGT, which has traditionally been applied primarily in the field of communication. Only in recent years has its application expanded to areas such as employee entrepreneurship and social media (Katz et al., 1973; Ali-Hassan et al., 2015; Zhang et al., 2018). This paper further extends the UGT framework to explore the use of GenAI tools in workplace contexts, empirically examining how cognitive and social motivations influence employees' use of these tools by enhancing their knowledge transfer and resource acquisition capabilities. In turn, this impacts their job satisfaction and innovative job performance. This extension not only significantly enriches the theoretical application of UGT but also offers a fresh perspective on understanding the mechanisms by which GenAI tools shape employee behavior.

Second, this study enriches the research perspective on the impact of GenAI tools on employee behavior. Existing literature on the relationship between GenAI tool usage and employee performance predominantly examines a single aspect of their use, often overlooking the underlying motivations and the complex mechanisms through which these tools influence performance (Kong et al., 2021; Tong et al., 2021; Chowdhury et al., 2022; Liang et al., 2024). By focusing on the motivations driving employees' use of technology, this paper identifies two primary categories of motivation: cognitive and social. Cognitive motivation arises from intrinsic needs such as knowledge acquisition, problem-solving, and skill enhancement, while social motivation pertains to the fulfillment of extrinsic needs like social interaction, building connections, and gaining a sense of belonging through technological tools. Through this perspective, the study offers a comprehensive examination of how cognitive and social motivations mediate the impact of GenAI tool usage on employees' innovative work performance, thereby providing a novel perspective on the relationship between GenAI and employee innovation.

Third, this study confirms the internal mechanisms underlying employee knowledge sharing, resource acquisition, and job satisfaction. It not only highlights the direct effect of GenAI tool usage on innovative job performance but also delves into the intricate connections between employees' knowledge-sharing and resource acquisition abilities and their job satisfaction, identifying these factors as key mediators in the relationship between GenAI tool use and innovative performance. Specifically, the use of generative AI tools driven by cognitive and social motivations indirectly boosts employee job satisfaction by enhancing knowledge transfer and acquisition capabilities, which subsequently fosters innovative job performance (Usmanova et al., 2021; Fischer & Döring, 2022; Wang & Yao, 2022; Yin & Niu, 2024; Wang, 2024; Gayathri & Bella, 2024; Amabile, 1988; Zhou & George, 2001; Zou et al., 2015). This theoretical framework not only clarifies how GenAI tools contribute to improving employee innovation performance but also provides a robust model for future research in this area.”

3. **Ethical approval process and data confidentiality are not adequately explained. Ethics committee approval or exemption statement for the research should be clearly stated; otherwise, it may pose a problem in terms of publication ethics.**

**Answer：**We sincerely thank the reviewers for the important reminder of the ethical compliance of the study. We have supplemented the relevant content according to the academic ethical norms, which are improved as follows:

Ethical review statement added: This study was approved by the Ethics Committee of Anhui Normal University in 2025 (approval number: AHNU-ET2025060) and followed the ethical guidelines of the Declaration of Helsinki throughout. All participants signed an informed consent form, explicitly aware of their right to withdraw at any time and that the data would be used for academic research only.

4. **One gets the impression that a large part of the article was created with a google translate translation. This negatively affects conceptual clarity and quality of expression. A professional English academic editing is required to make it suitable for publication.**

**Answer：**We sincerely thank the reviewer for their rigorous evaluation of the manuscript’s language quality and deeply apologize for the negative impact of expression ambiguities on conceptual clarity. To systematically address these concerns, we have implemented the following improvements:

1) Professional Academic Editing：Engaged certified academic editing services for sentence-level proofreading and rewriting of the full manuscript. Prioritized corrections to: Terminological precision, Syntactic logic, and academic convention compliance.
2) In the process of language refinement, the English expression of core concepts (e.g., “Innovative Job Performance”) is synchronized to ensure that it corresponds accurately to the concepts and the thesaurus of the Job Performance field.
5. **Practical contributions and recommendations should be developed and presented more clearly for practitioners. At the moment the suggestions are very general and can be expanded as suggestions.**

**Answer：**We have reworked the practical takeaways to provide relevant and practical advice from the perspective of individual employees and corporate management, respectively. From an organization's perspective, this paper provides valuable guidance on whether or not an organization should limit the use of generative AI tools by its employees. From the perspective of employees, this paper suggests that employees should be actively guided to use generative AI tools correctly and efficiently, so as to create a positive and innovative working atmosphere and realize a win-win situation for both enterprises and employees.

These revisions have been highlighted in blue in the revised manuscript.

“6.2 Practical implications

The findings of this study offer significant insights into whether organizations should impose restrictions on employee use of GenAI tools. On a positive note, the integration of GenAI tools within organizations proves highly effective. These tools address challenges such as file transfers in workplace settings and streamline daily tasks. Moreover, they uniquely enhance interpersonal communication and connections, dismantling information silos and fostering collaboration among team members. Additionally, employees' use of GenAI tools has a considerable impact on their job satisfaction, which is closely linked to their performance outcomes. Based on these findings, organizations should focus on establishing a high-quality technological environment that fully engages employees and encourages active use of these tools. The introduction of GenAI tools enables more efficient knowledge transfer and acquisition, providing employees with easy access to a wealth of resources that can expand their thinking and naturally bolster their innovation capabilities. Simultaneously, companies must continuously refine the tool’s functionality and user experience to better align with employees’ needs, thereby stimulating positive feedback and encouraging proactive engagement. This approach enhances individual performance and drives synchronized growth in overall organizational performance, leading to a mutually beneficial outcome for both employees and the organization.

The application of GenAI tools, as their integration into workplace environments expands, presents both opportunities and challenges. Enterprise management must explore the principles and business value of effectively utilizing GenAI tools to drive corporate performance improvements and achieve organizational objectives. Simultaneously, employees should be actively guided in the correct and efficient use of these tools to foster a positive, innovative work culture. This approach ensures a win-win outcome for both the enterprise and its employees, facilitating deep intellectual and emotional integration with their work—critical factors for the enterprise’s successful development. The growing prevalence of GenAI tools is an irreversible trend, making it impossible to return to a pre-GenAI era. Thus, the focus should be on fully harnessing their potential to support organizational growth, improving employee productivity by leveraging platforms like social media to unlock the substantial potential of these tools in the workplace.”

**Appendix**

1. **Some hypotheses lack strong theoretical justification. For example, hypothesis H2 is presented as follows:**

**“H2: The social use of GenAI tools positively impacts resource acquisition.” However, the direct relationship between social use and resource acquisition is not sufficiently explained. What kind of social interactions are linked to resource acquisition or how GenAI tools support these processes is not supported by literature. This relationship seems like an artificial connection and leaves the reader with a sense of theoretical emptiness.**

**Answer:** We sincerely appreciate your insightful critique regarding the theoretical grounding of our research hypotheses. In response, we have conducted thorough retrospection and literature verification, systematically strengthening the theoretical foundations across all hypotheses through additional scholarly evidence: For example, in the discourse of H2, we emphasize that the use of socially motivated generative AI tools based on social motivation enhances employees' access to resources, an idea that is derived from social exchange theory (Russell and Norvig, 2002). Furthermore, in addressing the above relationship, we provide relevant research evidence, for example, “Noy and Zhang (2023) found that the use of a generative AI tool, ChatGPT, significantly increased users' writing productivity and reduced time resources spent on the job” this evidence proves that “The use of generative AI tools based on social motivation can help employees to access all kinds of required knowledge resources thereby reducing the loss of resources for individual employees.”

All study hypothesis sections were re-stated and the revisions have been highlighted in blue in the revised manuscript.

“3.1 Cognitive use of GenAI tools and knowledge transfer

Knowledge transfer refers to the intentional exchange of explicit and implicit expertise between different organizational entities, facilitated by various communication channels (Spreitzer, 1996). Knowledge management frameworks illustrate that cognitive GenAI adoption accelerates knowledge acquisition in two key ways: by speeding up transactional processes and enhancing cognitive absorption (Autio & Nambisan, 2018). GenAI systems optimize the handling of informational repositories through algorithms that filter, semantically summarize, and retrieve content contextually (Gindert & Müller, 2024). For instance, Morgan Stanley utilized GPT-4 to process 100,000 company-specific investment documents and business materials, refining their knowledge assistance system for financial advisors (Gindert & Müller, 2024). Digital interfaces powered by GenAI technology transform complex information systems into more accessible, knowledge-friendly formats using adaptive data segmentation and connection methods (Autio & Nambisan, 2018). In practical applications, when employees engage in online meetings, GenAI tools can help generate meeting minutes, extract summaries from video or audio recordings, and organize key content—an application widely used on platforms such as ZOOM. Based on knowledge management theory, Zou et al. (2023) found that GenAI tools help break down interdepartmental knowledge barriers, fostering cross-disciplinary creative integration and enabling breakthrough solutions. Mu et al. (2023) further demonstrated that AI-assisted brainstorming tools significantly enhance team innovation output by generating diverse creative solutions. Thus, the following hypothesis is proposed:

H1: Cognitive use of GenAI tools positively impacts employees' knowledge transfer.

3.2 Social use of GenAI tools and resource acquisition capabilities

Workers require resource acquisition for their tasks, which includes access to information, materials, office space, and adequate working time. The acquisition of valuable resources within an organization originates from both internal elements and external sources, such as customers, suppliers, competitors, universities, government agencies, and technology intermediaries (Chen and Yang, 2014). The advent of GenAI tools enables the formation of informal relationship networks, in line with social exchange theory, by connecting individuals with shared interests and backgrounds, thereby expanding users' social networks (Russell and Norvig, 2002; Turner and Kuczynski, 2019). Organizational members are encouraged to promote collaborative tools, social sharing platforms, and interactive communication channels, as these facilitate team connections, enhance productive teamwork, and foster a sense of belonging among employees. GenAI tools, when utilized for social motivation, enable employees to access essential knowledge resources, reducing the strain on their personal resource usage. Noy and Zhang (2023) demonstrated that ChatGPT users experienced increased writing efficiency while significantly reducing their workplace time consumption. Jia et al. (2023) observed that AI technology implementation leads to better work distribution and increased employee creativity by alleviating human resource constraints. Zhan and Li (2024) highlighted that the informational advantage in a generative environment greatly enhances cognitive resource efficiency and situational awareness. Through GenAI tools, employees can more easily engage in communication, share information both within and outside the organization, and strengthen emotional connections with internal and external stakeholders. These social networks—comprising various internal and external members of the enterprise—provide a foundation of trust that supports employees' resource acquisition efforts, enhancing their ability to secure resources (Liu et al., 2019; Miao et al., 2024). Based on these insights, the following hypothesis is proposed:

H2: Social use of GenAI tools positively impacts employees' resource acquisition ability.

3.3 Knowledge transfer, resource acquisition, and job satisfaction

As previously discussed, knowledge transfer refers to the sharing of knowledge across organizational boundaries or between individuals, encompassing the dissemination of knowledge in various forms within or across different entities (Szulanski, 1996). Resource acquisition ability highlights employees' capacity to easily access relevant resources that facilitate their job tasks (Spreitzer, 1996). Employee job satisfaction is a multifaceted evaluation of work, representing the positive emotional state derived from the assessment of job experiences (Locke, 1976).

Knowledge transfer is a critical component of knowledge management, contributing to enhanced employee job satisfaction (Kianto et al., 2016; Zhou & Tang, 2017; Usmanova et al., 2021; Fischer & Döring, 2022). Knowledge transfer encourages employees to voluntarily share their work experiences, skills, and other job-related knowledge, which improves work efficiency (Usmanova et al., 2021; Fischer & Döring, 2022). For instance, Usmanova et al. (2021) found that knowledge-sharing behaviors among skilled employees in IT companies significantly contribute to their job satisfaction. Additionally, fostering a corporate culture that promotes knowledge transfer helps create a supportive interpersonal environment, strengthening work relationships and, consequently, enhancing job satisfaction (Borman & Motowidlo, 1997; Misuraca, 2013; Zhang et al., 2023). Research also suggests that knowledge sharing and transfer within organizations generate opportunities for concept development and job-related information acquisition, leading to greater job satisfaction (Misuraca, 2013; Zhou & Wei, 2011). Job satisfaction increases as knowledge transfer practices become more accessible within organizational environments, as indicated by Varshney and Damanhouri (2013). Based on these insights, the following hypothesis is proposed:

H3: Knowledge transfer positively impacts the improvement of employees' job satisfaction.

Previous research has established a positive relationship between resource acquisition capabilities and employees' job satisfaction (Mohr & Zoghi, 2008; Zatzick, 2011). Employees' resource acquisition ability is specifically demonstrated in their capacity to solve work-related problems, such as accessing external, tacit knowledge, obtaining additional resources to complete tasks, and other related factors (Spreitzer, 1996). A higher level of resource acquisition ability indicates that employees are more likely to enter a productive work state, maintain greater concentration, and exhibit enhanced cognition and assimilation of the organization's objectives, which ultimately leads to higher job satisfaction (Zatzick, 2011; Cheng & Wu, 2023; Xie et al., 2021). When employees adopt GenAI tools with an openness to change and innovation, coupled with a positive attitude, they can foster effective collaboration, strengthen their sense of identity and well-being, and improve their job satisfaction (Wang & Yao, 2022; Yin & Niu, 2024; Wang, 2024; Gayathri & Bella, 2024). For instance, Wang (2024) found that GenAI’s automated knowledge integration function reduces employees' information overload, allowing them to focus more on core tasks and, consequently, increasing job satisfaction. Gayathri & Bella (2024) highlighted that GenAI enhances employees' ability to quickly access both internal and external organizational resources. GenAI tools applied to social media platforms support content generation or improve user experiences, thereby boosting employees' familiarity with their work, improving their task completion abilities, and fostering more positive work attitudes, which contribute to greater job satisfaction. Based on these insights, the following hypothesis is proposed:

H4: Employee resource acquisition positively impacts the improvement of job satisfaction.

3.4 Job satisfaction and employees’ innovative job performance

Innovative job performance refers to employees’ work activities that go beyond conventional expectations, including the generation of creative ideas, problem-solving approaches, and the identification of new solutions (Katz, 1964; Scott & Bruce, 1994; Amabile et al., 1996). For businesses to thrive, enhancing competitiveness is critical, and competitiveness is driven by innovation. Thus, employees' innovative job performance is a core element in boosting organizational competitiveness (Yu & Shi, 2006; Liu & Li, 2025).

Extensive research indicates that high job satisfaction directly contributes to enhanced innovative performance. First, when employees are satisfied with their jobs, they tend to enjoy their work and exhibit greater willingness to perform (Boss, 1978). For instance, Schappe (1998) highlighted that higher job satisfaction leads to more positive perceptions of the organization, better work performance, and greater willingness to engage in behaviors beneficial to the organization. Xiao (2020) found that highly satisfied employees are more likely to internalize organizational goals, which in turn improves their innovative performance through organizational commitment. Second, job satisfaction is strongly and positively linked to the organizational innovation climate, which in turn positively influences employee innovation performance (Wang, 2011; Pang & Shi, 2016; Miao et al., 2024). For example, Yan and Bai (2016) found that a high-performance work system fosters a positive innovation climate, which enhances job satisfaction and boosts innovative behaviors. Finally, job satisfaction impacts employees' innovative job performance by influencing their emotional state, which affects their motivation and creativity (Amabile, 1988; Zhou & George, 2001; Zou et al., 2015). Amabile (1988) argued that positive affective states, such as job satisfaction, enhance cognitive flexibility and willingness to take risks, thereby increasing innovative output. Zhou and George (2001) suggested that job satisfaction indirectly promotes innovative behaviors by enhancing intrinsic motivation, such as interest in the task itself. Based on these findings, the following hypothesis is proposed:

H5: Employees' job satisfaction positively impacts the improvement of their innovative job performance.**”**

2. **A little more information about the** **pilot study could be added.**

**Answer:** Sincerely thank you for your valuable comments on this paper. In response to your suggestion that we need to add some information about the pilot study, we have added the reliability and validity of the pre-study data in 4.2 in the main text. The factor loadings of each variable were calculated by SmartPLS 4.0, and the combined reliability (CR=0.749-0.958), and validity (AVE=0.501-0.822) were greater than 0.5, which allowed us to proceed with the formal experiment.

Model aggregation validity analysis table

| Variable | Measurement item | Factor loading value | Composite Reliability | AVE value |
| --- | --- | --- | --- | --- |
| cognitive use of GenAI tools | CU 1 | 0.540 | 0.776 | 0.544 |
|  | CU 2 | 0.874 |  |  |
|  | CU 3 | 0.761 |  |  |
| social use of GenAI tools | SU 1 | 0.881 | 0.958 | 0.822 |
|  | SU 2 | 0.875 |  |  |
|  | SU 3 | 0.890 |  |  |
|  | SU 4 | 0.913 |  |  |
|  | SU 5 | 0.970 |  |  |
| knowledge transfer | know_tra1 | 0.318 | 0.784 | 0.501 |
|  | know_tra2 | 0.828 |  |  |
|  | know_tra3 | 0.661 |  |  |
|  | know_tra4 | 0.884 |  |  |
| resource acquisition | Acq_res1 | 0.845 | 0.872 | 0.694 |
|  | Acq_res2 | 0.852 |  |  |
|  | Acq_res3 | 0.800 |  |  |
| job satisfaction | Job_Stf1 | 0.824 | 0.941 | 0.763 |
|  | Job_Stf2 | 0.787 |  |  |
|  | Job_Stf3 | 0.900 |  |  |
|  | Job_Stf4 | 0.924 |  |  |
|  | Job_Stf5 | 0.924 |  |  |
| innovative work performance | I_Job_Pf1 | 0.614 | 0.749 | 0.503 |
|  | I_Job_Pf2 | 0.840 |  |  |
|  | I_Job_Pf3 | 0.653 |  |  |

**Special thanks to you for your good comments.**

**We tried our best to improve the manuscript and made some changes in the manuscript. These changes will not influence the content and framework of the paper. And here we did not list the changes but marked marked with different colors in revised paper. We appreciate for Editors/Reviewers’ warm work earnestly, and hope that the correction will meet with approval. Once again, thank you very much for your comments and suggestions.**

**Best wishes**

**Ayuan Zhang**

**18298257065@163.com**
