# Supplementary material for: The influence of generative artificial intelligence usage on employees’ innovative job performance
Source: PLoS One. 2026 Jan 22;21(1):e0327786. doi: 10.1371/journal.pone.0327786 (PMC12826493; doi:10.1371/journal.pone.0327786)
Supplement: Attachment — Submitted filename: response letter.docx [file pone.0327786.s006.docx]

**Response letter**

**Dear Editor and Reviewers,**

We sincerely appreciate the time and effort you devoted to reviewing our manuscript entitled ***“The Influence of Using Generative Artificial Intelligence Tools on Employee's Innovative Job Performance”* (Manuscript ID: PONE-D-25-16015).** We are truly grateful for your insightful comments and constructive suggestions, which have been invaluable in improving the clarity, rigor, and overall quality of our paper.

We have carefully revised the manuscript in accordance with all the comments provided by the editor and reviewers. Each suggestion has been thoughtfully considered, and corresponding changes have been made to strengthen the theoretical framework, refine the methodology, enhance the language, and enrich the discussion. **In the following sections, we provide a detailed, point-by-point response explaining how each comment has been addressed in the revised version.**

We deeply appreciate your constructive feedback and the opportunity to revise our work. Your guidance has greatly helped us enhance the scholarly contribution and readability of the manuscript.

**Editor**

|  | I secured the opinion of one reviewer, and I have carefully read the manuscript myself to offer my independent opinion. Both the reviewer and I believe that there are some merits in the study, and it may be of interest to readers of PLOS One upon major revisions. The authors should carefully consider each of my comments and the Reviewer’s comments. |
| --- | --- |
| **Reply** | We are very thankful for your acknowledgment of the study’s potential contribution and for your constructive suggestion to undertake major revisions. Following your guidance, we have carefully addressed each of your comments and those of the reviewer with comprehensive revisions throughout the manuscript. Specifically, we have refined the theoretical framework, clarified the definitions of key constructs, strengthened the methodological transparency, and enhanced the overall writing quality and logical flow. |
| **Comment 1** | In the Introduction section, it is not clear to me what is meant by “social use of AI”? Adding some examples would be useful, together with the use of example items in the Method section (see below). I have a similar concern regarding “resource acquisition” that could be better explained in the Introduction section. In the scale used, the resource acquisition is not explained to participants, with possible different interpretations by them. In general, since the topic is very new and in development, I think that the Introduction can be updated with more recent work (even published in 2025). |
| **Reply** | We sincerely thank the reviewer for this valuable comment and for highlighting the need to clarify the concepts of *“social use of AI”* and *“resource acquisition.”* In response, we have substantially revised the **Introduction** section to improve conceptual precision and to incorporate recent scholarly work (2023–2025).   1. **Clarification of “*social use of AI”*:** We have added a clear definition and practical examples to illustrate how employees utilize GenAI tools for social and collaborative purposes. The revised text now specifies that “social use involves using AI tools for collaboration, communication, and professional networking—such as co-creating content with colleagues via ChatGPT or coordinating cross-departmental tasks using AI assistants (Marimon et al., 2025).” 2. **Clarification of “resource acquisition”:** We have refined the definition to emphasize employees’ ability to obtain informational, material, and relational resources that support innovation. The revised passage reads: “this study refines the concept of resource acquisition, defined as employees’ ability to access informational, material, and relational resources that support innovation. GenAI facilitates this access through intelligent search, recommendations, and network-based collaboration tools (Lin & Wu, 2024).” 3. **Inclusion of updated literature:** Following your recommendation, we integrated recent studies from 2023–2025 (e.g., *Wang et al., 2023; Budhwar et al., 2024; Khan et al., 2025; Marimon et al., 2025*) to ensure the Introduction reflects the latest advancements in the field and strengthens the theoretical grounding of the manuscript.   These revisions have been implemented on ***Introduction*** of the revised manuscript. We believe these changes enhance conceptual clarity, theoretical relevance, and alignment with current research developments.  1. Introduction  Generative artificial intelligence (GenAI) refers to algorithms that autonomously generate original textual, visual, or audio content, mimicking human creativity and reasoning (Wang et al., 2023; Budhwar et al., 2024). Its adoption in the workplace has surged as organizations leverage GenAI to boost productivity, streamline decision-making, and foster innovation. The 2024 Microsoft Work Trend Index reports that over 90% of employees in China utilize GenAI for daily tasks, with more than half integrating AI tools into core business operations (Microsoft, 2024). This widespread adoption highlights GenAI’s potential to drive innovation and maintain a competitive edge (Khan et al., 2025).  Innovative job performance (IJP) encompasses employees’ proactive involvement in idea generation, problem-solving, and process improvement beyond routine tasks (Scott & Bruce, 1994). Recent research highlights the critical role of technological enablement and knowledge dynamics in shaping IJP within digital organizations (Ramadan et al., 2023; Wang et al., 2025). However, the impact of GenAI on innovation outcomes remains debated—some scholars argue that over-reliance on algorithms may hinder independent thinking (Sundar, 2020), while others suggest that responsible GenAI use fosters creativity and cross-disciplinary collaboration (Mingjie et al., 2024; Chandrasekera et al., 2025).  Building on Uses and Gratifications Theory (UGT), this study explores how employees utilize GenAI to satisfy cognitive and social needs that influence innovation. Cognitive use refers to leveraging GenAI for gathering and processing work-related knowledge, while social use involves using AI tools for collaboration, communication, and professional networking—such as co-creating content with colleagues via ChatGPT or coordinating cross-departmental tasks using AI assistants (Marimon et al., 2025). Both types of use promote collective intelligence and encourage innovative behavior. Furthermore, this study refines the concept of resource acquisition, defined as employees’ ability to access informational, material, and relational resources that support innovation. GenAI facilitates this access through intelligent search, recommendations, and network-based collaboration tools (Lin & Wu, 2024). By integrating cognitive and social motivations with resource acquisition, this research offers a contemporary framework for understanding how GenAI adoption enhances employees’ IJP in modern organizational environments. |
| **Comment 2** | In the Method section, the authors state that they target “employee groups”; what groups were targeted? How were the organizations selected? The type of organizations the participants work in should be included in the demographics. |
| **Reply** | We sincerely appreciate the reviewer for providing this valuable suggestion. The issue you raised regarding clarifying the organizational background of the participants is crucial for enhancing the external validity of the study. We fully agree that more detailed information about the types of organizations can enrich the context of our research. In response to your concerns, we have taken the following two specific measures:  We have revised the ***Method section (4.1.1 Questionnaire recipients)*** to more clearly articulate our broad sampling strategy and its alignment with the study's objective of capturing general patterns, rather than differences across specific organizational types. The revised text now reads:  4.1.1 Questionnaire recipients  This study employed a comprehensive sampling strategy, distributing surveys via the Credamo platform to office workers across China. The aim was to identify general patterns in the relationship between GenAI usage and innovative performance, rather than examining differences across specific organizational types. This approach enhances the reliability and generalizability of the findings to common workplace contexts.  In addition, to better explain the situation, we would like to provide you with more detailed background information: the Credamo platform we use is a professional online survey platform that maintains a large and diverse pool of participants, covering a wide range of industries, company sizes, and job functions. We adopt this nationwide sampling strategy precisely to obtain a holistic perspective on Chinese employees' use of GenAI. Although our demographic data do not specifically record participants' industries or organization types, sampling through this platform ensures that our sample is likely to include employees from multiple sectors such as technology, finance, education, manufacturing, and services. We believe that this broad sample base is appropriate and effective for the generalizable relationships our research aims to reveal.  2.More importantly, we have proactively acknowledged this as a specific limitation in the ***Revised Manuscript (Section 6.3 Limitations and Future Research)***. We have added the following sentence to the **fourth paragraph discussing sample limitations:**  Third, from a methodological perspective, this research primarily relied on self-reported survey data, which may introduce common-method bias. Future studies could enhance validity by incorporating multi-source or longitudinal data, such as supervisor assessments or objective performance indicators, and by employing hierarchical or multi-level modeling approaches. |
| **Comment 3** | “An explanation of workplace applications for GenAI tools was provided both at the start of the questionnaire and through screening questions to confirm participants' professional experience”. The authors should disclose this information and may include it in a Supplementary file. |
| **Reply** | We sincerely thank the reviewer for this valuable suggestion to enhance the transparency of our methodological procedures. In direct response, we have now prepared a Supplementary Material file titled “**Appendix**”.  This file contains: The exact introductory text presented to participants, which defined “workplace applications of GenAI tools” with concrete examples to ensure a common understanding. The specific screening questions used to verify participants' current employment status and their opportunity to use GenAI tools at work.  We believe this supplementary material fully addresses the reviewer's comment by providing a clear record of how we contextualized the study for participants and ensured the relevance of our sample. We have cited this supplementary file in the revised manuscript within ***Section 4.1.2 Questionnaire design***. *"*(The specific questionnaire content is shown in the appendix.)*"*  Instructions: In recent years, generative AI software (e.g., Doubao, Kimi, Chatgpt, DeepSeek, etc.) has been rapidly growing and penetrating various industries, with far-reaching impacts on work practices. Generative AI software not only helps employees retrieve work information quickly, but also optimizes information sharing by driving collaboration tools and platforms to promote team harmony and efficient collaboration. At the same time, generative AI software assists employees in creative conceptualization, stimulates innovative thinking, and promotes continuous innovation in work results. The wide application of generative AI software technology significantly improves work efficiency, optimizes workflow, provides strong support for team collaboration and innovation, and demonstrates its important value and indispensable role in the work field.  Screening Questions: Do you use generative AI software at work?  Yes.  No.(Questionnaire terminated) |
| **Comment 4** | In the descriptions of the scales, examples of items for cognitive and social use of AI are missing. Moreover, the response scales and range of values are missing for all instruments. |
|  | We sincerely thank the reviewer for this valuable and constructive feedback. The reviewer is absolutely correct in pointing out the need for greater detail regarding the measurement scales. We have thoroughly revised the manuscript to address these concerns, ensuring full transparency and replicability of our methodological approach.  Specifically, we have made the following revisions:   1. Specific item examples have been added: In the revised draft, under the subsections "***4.2.1. Cognitive use of GenAI tools"*** and "***4.2.2. Social use of GenAI tools,***" we have now included specific scale measurement items as examples.   4.2.1. Cognitive use of GenAI tools  Cognitive use of GenAI tools was measured using a modified version of Ali-Hassan et al.’s (2015) social media usage scale. Three items were included: "I use generative AI software (such as Doubao, KIMI, ChatGPT, DeepSeek, etc.) to collect work-related information and share content," "I use generative AI software ... to disseminate content at work," and "I access content created by others using generative AI software ..."  4.2.2. Social use of GenAI tools  Social use of GenAI tools was assessed using adapted items from Ali-Hassan et al.’s (2015) framework. This construct was measured with five items, such as: "I use generative AI software ... to build new relationships at work," "I use generative AI software ... to connect with people I wouldn’t meet at work," and "I use generative AI software ... to maintain close social connections with colleagues at work." (The full set of items is provided in the Appendix.)   2. Clarified the unified scale format: At the beginning of Section 4.2 'Variable measurement', we provided a more prominent and clear explanation of the scale format. We explicitly stated: "All constructs were measured using a 7-point Likert scale, ranging from 1 (strongly disagree) to 7 (strongly agree). " 3. Provided the complete questionnaire: As the reviewers have seen, we have submitted the complete survey questionnaire as an ***appendix (Appendix).*** This appendix contains all the measurement items for all constructs used in this study, presented clearly in a 7-point Likert scale format. It provides readers and reviewers with the most comprehensive information about the measurement instruments. |
| **Comment 5** | In the Results section, in Figure 2, a note should be added explaining the abbreviations used. |
|  | We sincerely thank the reviewer for this valuable comment. We agree that explaining the abbreviations will enhance the clarity and readability of ***Fig 2.*** Accordingly, we have now added a note to the figure caption that explicitly defines all abbreviations used in the path model. The revised caption now reads as follows:    ***Note:*** *CU = Cognitive Use of GenAI Tools; SU = Social Use of GenAI Tools; Know_tra = Knowledge Transfer; Acq_res = Resource Acquisition; Job_Stf = Job Satisfaction; I_Job_Pf = Innovative Job Performance.*  ***Fig 2. Results of structural equation modeling*** |
| **Comment 6** | Moreover, the information in Table 6 is not justified since the authors do not use these classifications in their results. Regarding the model’s results, they should be better presented. For instance, it seems that Figure 2 shows only total effects. Where can the reader find direct effects? |
|  | 1.We have now clearly and systematically applied the classification criteria from Table 6 in the mediation effect analysis. In the main text of '5.3 Mediation Effect Analysis,' based on the analysis results, we explicitly determined that the pathway from the use of cognitive GenAI tools to innovation performance falls under 'complementary mediation.' The pathway from the use of social GenAI tools to innovation performance falls under 'indirect-only mediation.' Meanwhile, in Table 7, Mediation Analysis, we added a new column 'Mediation Type,' in which we clearly labeled the types for the aforementioned two complete chain mediation pathways. 2.In the section ***"5.3 Mediation Effect Analysis***," we explicitly stated in the supplementary analysis of the direct paths that: "The use of cognitive GenAI tools has a significant direct positive effect on innovative work performance (β = 0.375, p < 0.001)." "The direct effect of using social GenAI tools on innovative work performance is not significant (β = -0.006, p = 0.928)." Clarifying the role of direct effects: We further explain that the results of these direct effect analyses are a key basis for determining the type of mediation (complementary mediation vs. indirect-only mediation). This allows readers to clearly see the values of the direct effects and their theoretical significance. The complete chained mediation paths in this study reveal two distinct types. First, for the cognitive use of GenAI tools (CU), a complementary mediation is observed. Cognitive use has a significant direct effect on innovation performance (β = 0.375, p < 0.001) as well as a significant indirect effect through knowledge transfer and job satisfaction (β = 0.082, p < 0.001). This suggests that cognitive use partially influences innovation performance through mediating mechanisms while also exerting an independent direct effect. Second, for the social use of GenAI tools (SU), an indirect-only mediation is found. The direct effect of social use on innovation performance is not significant (β = -0.006, p = 0.928), but the indirect effect through resource acquisition and job satisfaction is significant (β = 0.088, p < 0.001). This indicates that social use influences innovation performance entirely through mediating mechanisms. **Table 7. Mediator analysis** **Hypotheses** \| **Indirect effects** \| **t** \| **95% CI** \| **p** \| **Type of Intermediary**; **Cu→Know_tra→Job_Stf→I_Job_Pf** \| 0.082 \| 3.556 \| [0.037, 0.127] \| 0.000 \| Complementary mediation; **Cu→Know_tra→Job_Stf** \| 0.249 \| 6.454 \| [0.172, 0.326] \| 0.000 \| /; **Know_tra→Job_Stf→I_Job_Pf** \| 0.119 \| 3.606 \| [0.054,0.184] \| 0.000 \| /; **Su→Acq_res→Job_Stf→I_Job_Pf** \| 0.088 \| 4.153 \| [0.047, 0.129] \| 0.000 \| Indirect-only mediation; **Su→Acq_res→Job_Stf** \| 0.269 \| 7.151 \| [0.194, 0.344] \| 0.000 \| /; **Acq_res→Job_Stf→I_Job_Pf** \| 0.158 \| 4.384 \| [0.087, 0.229] \| 0.000 \| / ***Note:*** *This table only determines the type of mediation for complete chained mediation paths, as their direct effects (CU→CX and SU→CX) have been tested in the supplementary analysis. The mediation type of certain paths (e.g., CU→Know_tra→Job_Stf) is not determined because their corresponding direct effects (e.g., CU→Job_Stf) are not included as direct paths in the theoretical model, and thus the classification criteria in Table 6 cannot be applied.* |
| **Comment 7** | A very important point is that the writing is very poor and should be improved. In some parts of the manuscript, it is difficult to understand what is meant, especially in the Introduction and Discussion sections. I encourage authors to revise the manuscript with the aim of enhancing clarification. |
| **Reply** | We sincerely thank the reviewer for this valuable comment and for drawing our attention to the need for improving the overall clarity and readability of the manuscript. We fully acknowledge that certain parts of the earlier version—particularly the **Introduction** and **Discussion** sections—required significant refinement to improve linguistic precision and logical flow.  In response, we have undertaken a **comprehensive language revision** of the entire manuscript, with particular focus on:   1. **Rewriting and condensing the Introduction** to ensure smoother argumentation, clearer definitions (e.g., “social use of AI” and “resource acquisition”), and better integration of recent literature (2023–2025). 2. **Refining the Discussion section** to strengthen logical transitions, clarify theoretical implications, and ensure consistency with the results. 3. **Conducting professional English language editing** by a native English-speaking academic editor with expertise in management and organizational behavior, to ensure that grammar, syntax, and terminology meet international publication standards.   We believe these revisions have substantially enhanced the **clarity, coherence, and academic tone** of the manuscript, making the arguments easier to follow and the contributions more accessible to an international readership.  We greatly appreciate the reviewer’s constructive feedback, which has directly contributed to improving the overall quality and readability of the paper. |
| Comment 8 | Regarding the response to Reviewers, I have similar concerns; it was difficult for me to follow. For instance, in the Response to Reviewer 1 point 3, the authors’ citation is not consistent with what was then reported in the manuscript (in blue). |
|  | We sincerely thank the Editor for carefully reviewing both our manuscript and our previous response letter, and for pointing out the inconsistency between our response and the revised text in the earlier submission. We fully acknowledge that the citation discrepancy and insufficient alignment between the response explanation and the actual revision in the manuscript may have caused confusion.  In the current revision, we have **carefully rechecked every point-by-point response** to ensure that (1) all responses accurately reflect the corresponding revisions in the manuscript, (2) in-text citations are consistent with those referenced in the response letter, and (3) the modifications are clearly highlighted in the revised version.  Specifically, for **Reviewer 1 – Comment 3 (regarding causal inference and cross-sectional data)**, we have verified and refined the wording in both the ***Limitations and Future Research* section (paragraph 4)** and the response letter to maintain consistency. The revised section now explicitly reads:  “Third, from a methodological standpoint, this research primarily relied on self-reported survey data, which may introduce common-method bias. Future studies could strengthen validity by incorporating multi-source or longitudinal data, including supervisor assessments or objective performance indicators, and by applying hierarchical or multi-level modeling approaches.”  This wording is now fully aligned with the response text and citations provided in the revised version.  We sincerely appreciate the Editor’s attention to this matter, which prompted us to ensure absolute coherence and transparency between the manuscript and the response letter. These adjustments have improved both the **credibility and professionalism** of our resubmission. |

**Reviewer 2**

|  | Dear Authors,  Thank you for submitting the revised version of your manuscript. Your study presents a relevant and timely investigation into the factors influencing the adoption of AI-based technologies in healthcare settings in Saudi Arabia. The integration of TOE and UTAUT frameworks strengthens the theoretical foundation and helps capture both organizational and individual-level factors.  Your use of a mixed-methods design is well justified and effectively implemented. The quantitative data is analyzed appropriately through descriptive and regression analyses, while the qualitative interviews complement and reinforce the findings. The conclusions are supported by the data, and you have clearly acknowledged the limitations of your study.  The manuscript is generally well-structured and intelligible, although we recommend a professional language edit to address minor grammatical and stylistic issues, particularly in the discussion and conclusion sections.  Overall, this is a technically sound and methodologically robust contribution that addresses a significant research gap in digital health transformation, particularly in the context of developing economies.  Congratulations on your work. I wish you continued success in your future research endeavors.  Best regards. |
| --- | --- |
| **Reply** | We sincerely thank Reviewer #2 for the thoughtful and encouraging feedback. We truly appreciate your recognition of the study’s relevance, methodological rigor, and theoretical contribution to understanding the adoption and impact of AI-based technologies in organizational contexts.  In line with your valuable suggestion, we have undertaken a **comprehensive language revision** of the manuscript to enhance clarity, fluency, and stylistic precision—particularly in the **Discussion** and **Conclusion** sections. This revision was performed by a **professional academic editor who is a native English speaker** with expertise in management and technology research. The edits have improved sentence flow, coherence, and readability without altering the intended meaning of the original content.  We are deeply grateful for your positive assessment and kind encouragement. Your constructive input has been instrumental in refining both the linguistic quality and overall presentation of our manuscript. |

**Reviewer 3**

|  | Dear Editor  The attached article was checked. The manuscript contains interesting information about The Influence of Using Generative Artificial Intelligence Tools on Employee's Innovative Job Performance. I think that this article is well suited to your journal.  It is generally good work. The scientific and presentation level of the manuscript is high.  The title is understandable and in line with the text. The text is written in a descriptive and understandable language. The material and method are well described and adequately detailed Discussion and conclusion are interrelated. |
| --- | --- |
| **Reply** | We sincerely thank Reviewer #3 for the positive and encouraging evaluation of our manuscript. We are truly grateful for your kind recognition of the study’s relevance, clarity of presentation, and methodological rigor.  Your feedback affirms that our efforts to develop a coherent, well-structured, and practically meaningful investigation of generative AI’s influence on employees’ innovative job performance have been successful. We have made further minor refinements to enhance linguistic accuracy and coherence across the manuscript, ensuring that the content remains clear and accessible to a broad readership.  We deeply appreciate your supportive comments and your affirmation that the paper is suitable for publication in this journal. Your positive evaluation motivates us to continue developing high-quality research in this emerging field. |

**Reviewer 4**

| **Comment 1** | A few of the sentences in the intro and discussion are clumsy or wordy and could be helped by some light language editing for concision and clarity. |
| --- | --- |
| **Reply** | We sincerely thank Reviewer #4 for this helpful observation. We fully agree that the readability of certain parts of the manuscript—particularly in the **Introduction** and **Discussion** sections—could be improved through more concise phrasing and smoother sentence structure.  Accordingly, we have carefully revised these sections to enhance **clarity, brevity, and flow**. Redundant expressions were removed, and long or complex sentences were simplified without altering the intended meaning. Additionally, the entire manuscript underwent **a professional English language edit** conducted by a native English-speaking academic editor specializing in management and organizational research.  We believe these revisions have significantly improved the manuscript’s readability, precision, and academic tone. We are very grateful for your constructive recommendation, which helped us strengthen the overall presentation quality of the paper. |
| **Comment 1** | Figure 1 (conceptual model) could be redrawn for easier visual clarity. For example, use the same shapes and even spacing to make it easier to read. |
| **Reply** | We are grateful to the reviewer for this valuable comment regarding the presentation of our conceptual model. Following the suggestion, we have thoroughly redesigned Figure 1 to enhance its visual clarity. The revisions include:   1.**Standardizing Shapes:** We have used consistent geometric shapes for all model constructs to create a uniform visual language. 2.**Optimizing Layout:** We have meticulously adjusted the spacing and alignment of all elements to achieve a balanced and well-organized structure. 3.**Improving Readability:** The overall readability has been significantly improved, making the proposed relationships in the model easier to follow.   We believe these changes have greatly improved the figure, and we thank the reviewer again for the constructive feedback.    **Fig 1.Theoretical framework** |
| **Comment 2** | You could mention ethical implications of GenAI use in the workplace (e.g., privacy, bias, algorithmic transparency) briefly, even speculatively. |
| **Reply** | We are grateful to the reviewer for this insightful suggestion. As advised, we have incorporated a discussion on the ethical dimensions of workplace GenAI implementation in the ***6.3 Limitations and future research section***  **(paragraph 5)**. We now briefly mention key challenges like data privacy, algorithmic bias, and algorithmic transparency, and speculatively link them to employees' trust and innovative behaviors. We also propose the examination of ethical AI management frameworks as a direction for future inquiry. This valuable comment has helped us broaden the perspective of our manuscript.  Finally, the ethical considerations surrounding GenAI use in the workplace warrant further investigation. Issues such as data privacy, algorithmic bias, and transparency in AI-assisted decision-making raise important questions regarding fairness and accountability. Employees' perceptions of ethical AI governance may influence their trust in technology and their willingness to engage in innovation. Future research could explore how ethical AI management frameworks—such as transparent model explanations or privacy-by-design practices—shape employees' attitudes, psychological safety, and creative performance. |

**Editor**

| **Comment 1** | Please ensure that you include a title page within your main document. You should list all authors and all affiliations as per our author instructions and clearly indicate the corresponding author. |
| --- | --- |
| **Reply** | Thank you for your suggestion. We have created the title page based on the sample file from PLOS ONE. The title also lists all authors and all affiliations, with the corresponding author clearly indicated.  **Title：The Influence of Generative Artificial Intelligence Usage on Employees’ Innovative Job Performance**  Hui Zhang1, Lidong Zhu1, Ayuan Zhang1*,Kilichov Shohruh1  1School of Economics and Management, Anhui Normal University, Wuhu, Anhui, China.  **Corresponding author.** *Ayuan Zhang,  **Corresponding author address:** No.189, Jiuhua South Road, Wuhu City, Anhui Province, Huajin Campus of Anhui Normal University.  **Corresponding author e-mail:** 18298257065@163.com. **Tel:** 86-18298257065. |
| **Comment 2** | Please amend either the abstract on the online submission form (via Edit Submission) or the abstract in the manuscript so that they are identical. |
| **Reply** | Thank you for your comment. We have amended the abstract in the online submission system via the 'Edit Submission' feature to ensure it is now identical to the abstract in the manuscript file. |
| **Comment 3** | Please remove your figures/ from within your manuscript file, leaving only the individual TIFF/EPS image files. These will be automatically included in the reviewer’s PDF. |
| **Reply** | Thank you for this guidance. We have now removed all figures from within the manuscript file, as requested. The individual TIFF/EPS figure files have been uploaded separately via the submission system. All figure captions remain in their correct places within the manuscript to guide the reviewers.  We believe the submission now fully complies with the journal's technical requirements. |
| **Comment 4** | Please ensure that you refer to Table 7 in your text as, if accepted, production will need this reference to link the reader to the Table. |
| **Reply** | Thank you for your important reminder. We have now ensured that Table 7 is correctly referenced in the main text. Specifically, we added a reference to Table 7 in the section on the analysis of specific mediating paths to guide readers. The revised manuscript now fully meets this requirement.  The complete chained mediation paths in this study reveal two distinct types. First, for the cognitive use of GenAI tools (CU), a complementary mediation is observed. Cognitive use has a significant direct effect on innovation performance (β = 0.375, p < 0.001) as well as a significant indirect effect through knowledge transfer and job satisfaction (β = 0.082, p < 0.001). This suggests that cognitive use partially influences innovation performance through mediating mechanisms while also exerting an independent direct effect. Second, for the social use of GenAI tools (SU), an indirect-only mediation is found. The direct effect of social use on innovation performance is not significant (β = -0.006, p = 0.928), but the indirect effect through resource acquisition and job satisfaction is significant (β = 0.088, p < 0.001). This indicates that social use influences innovation performance entirely through mediating mechanisms (Table 7). |
| **Comment 5** | Thank you for including your ethics statement on the online submission form: "Human Subject Research Anhui Normal University AHNU-ET2025060".  To help ensure that the wording of your manuscript is suitable for publication, would you please also add this statement at the beginning of the Methods section of your manuscript file. |
| **Reply** | Thank you for your guidance regarding the ethics statement. We have complied with your request and added the full ethics declaration at the beginning of the Methods section (please see section 4.1.1 "Questionnaire recipients" in the manuscript file).  The added statement is as follows:  "This study involving human subjects was reviewed and approved by the Ethics Committee of Anhui Normal University (Approval No: AHNU-ET2025060). Written informed consent was obtained from all participants."  We confirm that the wording in the manuscript now matches the declaration provided on the submission form. Thank you for ensuring the clarity and compliance of our work. |
