# Supplementary material for: The influence of generative artificial intelligence usage on employees’ innovative job performance
Source: PLoS One. 2026 Jan 22;21(1):e0327786. doi: 10.1371/journal.pone.0327786 (PMC12826493; doi:10.1371/journal.pone.0327786)
Supplement: Attachment — Submitted filename: response_letter_auresp_3.docx [file pone.0327786.s007.docx]

**Response letter**

**Dear Editor ,**

We sincerely appreciate the time and effort you devoted to reviewing our manuscript entitled ***“The Influence of Using Generative Artificial Intelligence Tools on Employee's Innovative Job Performance”* (Manuscript ID: PONE-D-25-16015).** We are truly grateful for your insightful comments and constructive suggestions, which have been invaluable in improving the clarity, rigor, and overall quality of our paper.

We have carefully revised the manuscript in accordance with all the comments provided by the editor. Each suggestion has been thoughtfully considered, and corresponding changes have been made to strengthen the theoretical framework, refine the methodology, enhance the language, and enrich the discussion. **In the following sections, we provide a detailed, point-by-point response explaining how each comment has been addressed in the revised version.**

We deeply appreciate your constructive feedback and the opportunity to revise our work. Your guidance has greatly helped us enhance the scholarly contribution and readability of the manuscript.

**Editor**

|  | I carefully read the authors' responses to my comments and the reviewers' feedback. I believe that the authors did a good job in responding to each point. For this reason, I consider the manuscript accepted upon minor revisions. |
| --- | --- |
| **Reply** | Thank you for your reminder. We have now included the Appendix in the revised manuscript, placed after the References section. The Appendix contains the complete survey instrument used in this study, including all measurement items for the six constructs (cognitive use, social use, knowledge transfer, resource acquisition, job satisfaction, and innovative job performance). This addition enhances the transparency and reproducibility of our research. |
| **Comment 1** | In particular, I kindly request that the authors submit the Appendix file, which is currently unavailable on the platform. Moreover, regarding response to Reviewer 1, Comment 3, I believe that in the modified insert in the manuscript, the reference to the important limitation regarding the cross-sectional nature of the data and the impossibility of establishing causal inference is now missing. |
| **Reply** | We sincerely apologize for this oversight and thank you for highlighting this critical methodological point. We fully agree that the cross-sectional nature of our data represents a significant limitation for establishing causal directions. In direct response to your comment, we have thoroughly revised the “Limitations and Future Research” section (Section 6.3) to explicitly and prominently address this issue.  1.We have added a dedicated new paragraph as the first point in Section 6.3 to immediately foreground this key methodological constraint. This paragraph clearly articulates the limitation and proposes a concrete direction for future research to overcome it.  2.We have reorganized the structure of Section 6.3 to improve its logical flow. By presenting methodological limitations (cross-sectional design, common-method bias) first, followed by sample and theoretical limitations, the section now builds a more coherent argument about the study's boundaries.  3.The revised text integrates your suggestion by not only stating the limitation but also outlining how future research can employ more robust designs to establish causality.These revisions have been implemented on Introduction of the revised manuscript. We believe these changes enhance conceptual clarity, theoretical relevance, and alignment with current research developments.  6.3 Limitations and future research  While this study confirms the positive impact of GenAI tools on employees' innovative job performance, several limitations remain, offering avenues for further exploration.  First, from a methodological perspective, the cross-sectional design limits the ability to establish causal inferences. Although the hypothesized relationships are grounded in theoretical reasoning and empirical support, the reliance on a single time point of data collection prevents definitive conclusions about the direction of causality. Future research could employ longitudinal or experimental designs to better capture the dynamic and causal nature of the relationships examined.  Second, the research primarily relied on self-reported survey data, which may introduce common-method bias. To enhance validity, future studies could incorporate multi-source data (e.g., supervisor ratings, objective performance indicators) or longitudinal approaches, and employ statistical techniques such as hierarchical or multi-level modeling to mitigate potential biases.  Third, the sample primarily reflects general enterprise employees, 90% of whom hold at least a bachelor's degree. This limits the generalizability of the findings across industries, job levels, and educational backgrounds. Future research could investigate whether the effects of GenAI vary across sectors such as healthcare, finance, or manufacturing, as well as across different managerial hierarchies and regional contexts. Additionally, personal factors—such as educational attainment, openness to experience, and digital literacy—may moderate the influence of GenAI on innovation outcomes. Exploring these moderating effects could provide organizations with more targeted guidance for employee-focused AI strategies.  Fourth, while this study highlights the positive indirect impact of cognitive and social GenAI use on IJP, it does not address potential negative outcomes. Overreliance on GenAI tools may lead to issues such as information overload, reduced cognitive engagement, or "social saturation," where constant digital interaction hinders creativity. Future research should examine both the enabling and constraining effects of GenAI use to capture its dual influence on innovation more comprehensively.  Finally, the ethical considerations surrounding GenAI use in the workplace warrant further investigation. Issues such as data privacy, algorithmic bias, and transparency in AI-assisted decision-making raise important questions regarding fairness and accountability. Employees' perceptions of ethical AI governance may influence their trust in technology and their willingness to engage in innovation. Future research could explore how ethical AI management frameworks—such as transparent model explanations or privacy-by-design practices—shape employees' attitudes, psychological safety, and creative performance.  In conclusion, while this study provides empirical evidence of GenAI's beneficial role in fostering innovative job performance, future investigations should adopt a broader, ethically informed, and methodologically diverse approach to fully understand the complexities of AI-driven innovation in organizational contexts. |
